# Hospital News

**Published:** 1922-08

**Authors:** 


### HOSPITAL NEWS.

The new memorial wing, costing £21,000, has been opened at Stroud General Hospital.

The private nursing staff, beginning with four nurses, is being re-established at Hereford General Hospital.

Sir Charles Brown has published his recollections in a book called "Sixty-Four Years a Doctor." The proceeds will benefit the Preston Royal Infirmary.

Standish House, Stonehouse, Glos, has been opened as a sanatorium for 150 patients by the County Joint Committee for Tuberculosis.

The new wing at Ripley Cottage Hospital has been formally opened. It contains 12 beds, and has cost £3,000. The hospital is free from debt.

Mr. and Mrs. A. C. Pain have offered to give a new operating theatre in memory of their son, Capt. E. D. Pain, to the Frimley and District Cottage Hospital. An X-ray room, a store-room and an extra servant's bedroom are also needed.

A silver salver and a cheque have been presented to Dr. Latimer Greene, on his retirement from the post of honorary medical officer to the Stratford-on-Avon Hospital after forty years' service.

Bretby Hall, acquired by the Derbyshire County Council for an open-air school for tubercular and crippled children, is to be sold, since the Council cannot afford to carry on the scheme.

At Fazakerley, the largest of the three hospitals controlled by the Liverpool Corporation, is a school for tuberculous children, about fifty of whom are taught daily by a trained and certificated teacher.

The old prison at Barnstaple, which has been converted into a hospital for infectious diseases, is not found satisfactory, and the medical officer of health regrets that a joint hospital is not provided for a combined district.

Next year the Ulverston and District Cottage Hospital will celebrate its jubilee. The object of the celebrations will be a new hospital, since neither the size nor the situation of the original building is equal to the present volume of work.

The Slough Nursing Fund and the Slough War Memorial Committee have approved a scheme to build a home for the nurses on a central site, capable of extension. The trustees will include representatives of both and of the trustees of the present Nurses' Home.

The North Middlesex Hospital, formerly Edmonton Infirmary, now receives school-children and private patients, including those in its Maternity Home, which is reached by a separate entrance. Its special departments include a new theatre, orthopædic massage, and X-ray rooms.

The Lady Winchester Hospital and the New Sussex Hospital for Women and Children, Brighton, have benefited from a bazaar held at Roedean, the famous girls' school, where most of the articles, including pottery and furniture, were the work of the pupils. The school has its own kiln and carpenter's workshops.

The Foyle Intercepting Hospital, for sea-borne infectious diseases on ships arriving at Lough Foyle, which has been built by the Derry Port Sanitary Authority, has been formally opened. The hospital, costing £3,000, contains two wards, with accommodation for six patients, the staff, and a mortuary, washhouse and incinerator.

The official opening of Lostock Hall Convalescent Hospital for women and children, patients from Preston Royal Infirmary, completes the generous gift made by the chairman, Lieut.-Commander Harry Dewhurst, M.P., and Messrs. G. &. R. Dewhurst, Ltd., of Preston. There are three wards for women and two for children, and 50 patients can be accommodated. The architect is Mr. A. C. M. Lillie. The donor spent the first twelve years of his married life at Lostock Hall.

The new children's and women's wards at St. John's Hospital, Lewisham, are expected to be opened at the end of the summer.

The Wharncliffe Pensions Hospital has reverted after seven years to its pre-war purpose, and has become once more Wadsley Asylum.

Miss M. D. M. Petre has given Mulberry House, Storrington, Sussex, for a hospital. Patients will contribute according to their means. Mr. E. J. Greenfield is hon. secretary, and Sister Annie Sibley matron.

The police refused to permit a barrel-organ collection for St. John's Hospital, Twickenham. The organiser, Mr. G. H. Shortt, complains that the organs dragged through the city by students of the larger hospitals were not interfered with, though, he says, no permit was even asked for.

The Royal Southern Hospital Ladies' Linen League has contributed twice the number of articles provided in 1921—namely, 2,325. The Ladies' Linen League was inaugurated in 1911 by Mrs. Lyon H. Maxwell (honorary treasurer), and was the first of its kind in Liverpool.

Additional accommodation for ten nurses in a separate building, and a new operating theatre with marble walls and floor, have been added to Eaton Hospital, Middlesbrough. In the latter all the fittings are movable. Mr. J. A. Lofthouse, A.R.I.B.A., is the architect.

The British colony in Spain and the Canary Islands has raised a fund to supply the mechano-therapy rooms at the Red Cross Hospital, Madrid, with the necessary instruments. The staff, says the "Times," correspondent, has brought great relief to the wounded from Morocco, and the equipment is the first of its kind to reach Spain.

To commemorate the first investigation and treatment in this country of sleeping-sickness, Mr. T. H. Openshaw, lately senior surgeon at the London Hospital, has asked that a tablet be placed over the bed formerly occupied by an African chief called Mandombi, who volunteered for experimental treatment.

The Wrenbury Hall Tuberculosis Training Colony, Cheshire, purchased by the Joint Committee of the British Red Cross Society and the Order of St. John, as a memorial of their work in the county, has been handed over to the County Council. The colony possesses 170 acres of land, and is part of the county scheme for treating tuberculosis.

On August 1, the Homœpathic and General Hospital, Plymouth, will reopen, after having been closed for six weeks to allow the quick installation of a central heating system. The 17 in-patients were discharged on June 17, and, in the meantime, accident cases have been received at the South Devon and East Cornwall Hospital, or at the Royal Albert Hospital, Devonport. The staff has taken its summer holiday, and this is the first occasion for the hospital to close since its opening fifty years ago.

It has been decided to amalgamate the Norfolk and Norwich Eye Infirmary with the Norfolk and Norwich Hospital in a scheme which will allow the Eye Infirmary to retain its identity. Thus ends a movement which began in 1904, when a proposal to establish an eye department at the Norfolk and Norwich Hospital led to the idea of fusion with the Eye Infirmary. In 1908 the governors approved the scheme of amalgamation, but it was not till two years later that the Charity Commissioners issued a final draft. In 1912 the present premises were purchased by the Hospital out of funds allotted for eye cases. In 1913 the Eye Infirmary moved from Pottergate Street, and in the July following the Duke of Norfolk opened the present buildings by the Fountain. The pressure of circumstances continued to encourage amalgamation, which, it is believed, while preserving the identity of the Eye Infirmary, will mean a substantial economy, and a useful extension of its work.

